# Analysis of MRI Artifacts Induced by Cranial Implants in Phantom Models

**DOI:** 10.3390/healthcare13070803

**Published:** 2025-04-03

**Authors:** Bibiána Ondrejová, Viktória Rajťúková, Kristína Šavrtková, Alena Galajdová, Jozef Živčák, Radovan Hudák

**Affiliations:** 1 Department of Biomedical Engineering and Measurement, Faculty of Mechanical Engineering, Technical University of Košice, 042 00 Košice, Slovakia; viktoria.rajtukova@tuke.sk (V.R.); jozef.zivcak@tuke.sk (J.Ž.); radovan.hudak@tuke.sk (R.H.); 2 Department of Industrial Automation and Mechatronics, Faculty of Mechanical Engineering, Technical University of Košice, 042 00 Košice, Slovakia; alena.galajdova@tuke.sk

**Keywords:** implants, MRI imaging, artifacts, phantom models

## Abstract

**Background/Objectives**: Cranial reconstruction (cranioplasty) is a surgical procedure performed to restore skull function and aesthetics following trauma, oncological conditions, or congenital defects. Magnetic resonance imaging (MRI) is commonly used for the postoperative monitoring and diagnosis of patients with cranial implants. However, MRI artifacts caused by these implants can compromise imaging accuracy and diagnostic precision. This study aims to evaluate the extent of MRI artifacts caused by titanium and polyether ether ketone (PEEK) cranial implants and to identify optimal imaging sequences to minimize these artifacts. **Methods**: Phantom skull models with cranial defects of varying sizes (one-quarter, one-third, and one-half of the skull) were used to simulate real-world clinical conditions. The defects were filled with a water-based medium containing simulated brain tissue and tumor models. Custom 3D-printed titanium and PEEK cranial implants were fixed onto the phantom skulls and scanned using 1.5 T and 3 T MRI scanners. Various imaging sequences were tested, with a focus on optimizing parameters to reduce artifact formation. Turbo Spin Echo (TSE) sequences with fat saturation were implemented to assess their effectiveness in artifact reduction. **Results**: The study found that MRI artifacts varied based on the implant material, defect size, and magnetic field strength. A higher field strength (3 T) resulted in more pronounced artifacts. However, the use of TSE sequences with fat saturation significantly reduced artifacts and improved lesion visualization, enhancing diagnostic accuracy. **Conclusions**: This research highlights the importance of optimized MRI protocols when imaging patients with cranial implants. Proper selection of imaging sequences, particularly TSE with fat saturation, can mitigate artifacts and improve diagnostic precision, ultimately benefiting patient outcomes in clinical radiology.

## 1. Introduction

Magnetic resonance imaging (MRI) is a cornerstone of modern neuroimaging, providing high-resolution visualization of cranial structures without ionizing radiation. However, the presence of metallic cranial implants introduces significant imaging challenges due to the formation of artifacts, which can obscure critical anatomical details and reduce diagnostic accuracy. These artifacts arise from magnetic susceptibility differences between the implant material and surrounding tissue, leading to signal voids, distortions, and geometric deformations in MRI scans [1].

Cranial implants are commonly used in neurosurgical procedures such as cranioplasty, tumor resection, and trauma repair. These implants are primarily composed of titanium, polyetheretherketone (PEEK), or composite materials, each exhibiting different MRI artifact profiles. Titanium, widely regarded for its biocompatibility and mechanical strength, often produces extensive susceptibility artifacts, whereas CFRP (carbon fiber-reinforced polymer) implants demonstrate reduced MRI interference [2].

Recent advancements in MRI artifact reduction techniques have focused on sequence optimization, including the use of Turbo Spin Echo (TSE), View Angle Tilting (VAT), and Slice Encoding for Metal Artifact Correction (SEMAC) to enhance image quality in the presence of implants [3]. Additionally, research employing phantom models—artificial skull replicas designed to mimic human cranial structures—has proven essential for systematically evaluating implant-induced MRI artifacts under controlled conditions [4].

This study analyzes the impact of cranial implants on MRI artifact formation using phantom models. By evaluating different implant materials, sizes, and imaging sequences, we aim to quantify artifact severity and improve diagnostic accuracy. This study hypothesizes that artifact severity will vary based on the implant material, size, and MRI field strength, with a higher field intensity (3 T) causing more pronounced distortions.

## 2. Materials and Methods

**Figure 1:**
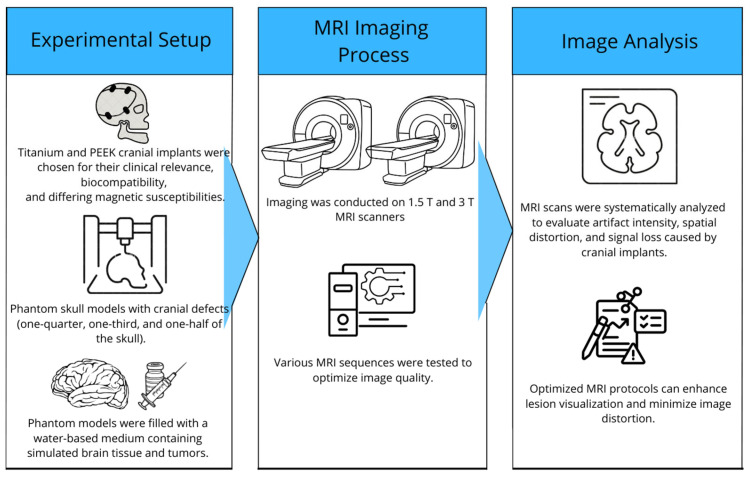
Overview of the study of MRI artifacts induced by cranial implants.

In this study, phantom skull models were used to create a realistic and controllable representation of the human skull. For imaging comparison, phantom skull models were developed with a density similar to that of brain tissue, incorporating 3D-printed cranial implants and tumor simulation (Figure 1).

### 2.1. Preparation of Phantom Models

**Figure 2:**
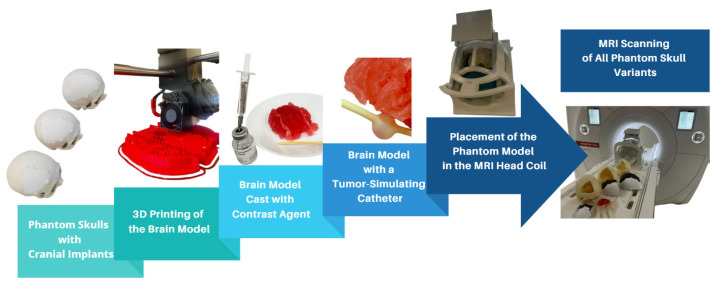
Visualization of the complete MRI scanning setup, including 3D-printed skulls with implants, brain model casting, tumor simulation, and placement within the MRI head coil.

The brain model was constructed from six individual parts, which, when assembled, formed a mold used for casting a prepared filling material. To refine the STL (Standard Triangle Language) models, the ideaMaker 4.3.1 software (RAISED 3D E2, USA) was employed. For the 3D printing process, the RAISE3D Pro2 Plus printer was utilized with PLA (polylactic acid) filament as the material. A total of 91.7 g (30.74 m) of PLA was consumed, and the printing duration totaled 24 h. Printing was conducted in two separate runs, each producing three segments of the model. After printing, the supports were removed, and a casting mixture was prepared to achieve a density similar to that of brain tissue. The mixture consisted of gelatin, glycerol, sugar, and food-grade dye, processed under boiling conditions to form a thick, homogeneous liquid. This mixture was then poured into the 3D-printed brain mold and cooled until it solidified (Figure 2). After several hours, the mold was removed, yielding an anatomically shaped brain.

To accommodate the varying sizes of cranial implants, the fabricated brain model was trimmed to fit the specific openings in the phantom skulls. A simulated tumor, represented by circular forms filled with gadolinium-based contrast agent, was embedded in each section of the gelatin-based brain model.

### 2.2. Application of Contrast Agent

A gadolinium-based contrast agent (1.0 mmol/mL) was used to simulate tumor presence (Figure 2) and enhance lesion visualization within the phantom models. The contrast agent was enclosed in a balloon catheter system to maintain stability throughout the imaging process and prevent diffusion-related signal variations. This setup ensured consistent artifact evaluation while allowing for differentiation between implant-induced artifacts and actual anatomical structures. Given the paramagnetic properties of gadolinium, the contrast agent improved the signal intensity by shortening the T1 and T2 relaxation times, thereby enhancing the visibility of simulated lesions in MRI scans. Additionally, the balloon catheter method used to introduce the contrast agent ensured a controlled and repeatable simulation of contrast uptake, allowing for direct comparisons between different implant materials and MRI sequences.

### 2.3. Imaging of the Phantom with Magnetic Resonance

The phantom models were imaged using two MRI devices. The first was the 1.5-tesla Magnetom Altea and the second device was the 3-tesla Magnetom Vida. The phantom models were supplemented during imaging with a water-based filling to enable the MRI scanner to generate resonance; without this, the sequences could not be initiated. The water was contained in disposable medical gloves, which were carefully positioned inside each phantom skull to cover as much internal surface area as possible. To ensure the visualization of the skull edges, cloth towels were soaked in water and affixed to the outer surfaces of the models.

The simulated tumor (a catheter filled with contrast agent) was adjusted in depth to monitor the formation of artifacts and assess its visibility. The skulls were imaged in sections, with each section scanned using implants made from titanium and PEEK materials. The imaging sequences were selected by a radiologist based on prior experience with tumor and cranial implant visualization. Image reconstruction after sequence acquisition was not feasible; therefore, parameters to minimize artifact formation had to be adjusted before starting each sequence. For each skull, two to three sequences were chosen. The total imaging time for a single skull was limited to a maximum of 20 min. For each sequence (Table 1), the field of view (FoV) was adjusted, phase encoding was set, and image contrast was optimized by suppressing either fat or water to enhance visualization. The slice thickness and resolution matrix remained unchanged, as modifications would not contribute to improved imaging quality.

The adjustments to imaging parameters before acquiring each sequence were based on predefined optimization criteria aimed at reducing artifact severity while maintaining image clarity and diagnostic relevance. These criteria were determined by a combination of the prior literature, pilot scans, and real-time artifact assessment. The key factors influencing parameter adjustments included artifact severity and distribution, signal-to-noise ratio (SNR) optimization, contrast enhancement and suppression strategies, spatial resolution and coverage, and field strength considerations (1.5 T vs. 3 T).

The sequence duration varied depending on the FoV size, which determined the region of interest.

1. Implant Material: Specifies the material of the cranial implant used in the study, either titanium or PEEK, each selected for its unique interaction with MRI imaging.
2. Implant Coverage: Indicates the proportion of the phantom skull covered by the implant, categorized as Type 1 (one-quarter), Type 2 (one-third), or Type 3 (one-half). This parameter allows for the evaluation of artifact formation and imaging quality relative to the size of the implant.
3. Sequence: Lists the MRI imaging sequences employed during the study. Each sequence is tailored to optimize visualization of the phantom model and reduce artifacts, such as those caused by implants or surrounding structures.
4. Slice Thickness [mm]: Refers to the thickness of the individual imaging slices captured during the sequence, measured in millimeters. This parameter affects the resolution and detail of the final image.
5. Sequence Duration [min]: Specifies the time required to complete each imaging sequence, measured in minutes. The duration may vary depending on the field of view (FoV) or additional imaging adjustments.
6. Contrast: Describes the type of contrast enhancement or suppression applied during the imaging sequence, such as fat suppression, water suppression, or SPAIR (Spectral Attenuated Inversion Recovery).
7. Resolution Matrix [mm]: Denotes the resolution of the resulting images, represented as a pixel grid. Higher resolution matrices provide greater detail in the final image.

## 3. Results

The primary objective of this study was to determine whether cranial implants influence the formation of imaging artifacts, obscuration of brain structures, or the visibility of tumors during MRI examinations. The generation of MRI artifacts differs significantly from those observed in CT imaging due to multiple influencing factors, including the imaging system, scanning methodology, and external conditions. The evaluation of artifacts was conducted through qualitative analysis and assessed by a qualified radiologist.

### 3.1. Types and Causes of MRI Artifacts

MRI artifacts can arise due to system-related factors, imaging techniques, or external interferences. Among system-induced artifacts, the most common include image inhomogeneity and radiofrequency (RF) pulse inhomogeneity. These issues can lead to localized or global signal loss, which is often caused by improper magnet calibration (e.g., shortening of the Free Induction Decay (FID) and Spin Echo (SE)), metallic objects, or susceptibility differences between materials.

A frequent source of artifacts in MRI is local inhomogeneity of the main magnetic field, which can be easily recognized in images. While incorrect magnet calibration is now rare due to technological advancements, issues persist in the peripheral regions of large fields of view (FoVs). This is primarily because modern MRI systems utilize shorter magnets with wider bores, making it increasingly challenging to maintain perfect field homogeneity in these regions.

The presence of metallic implants significantly disrupts the homogeneity of the magnetic field, leading to signal voids, image distortion, and susceptibility artifacts. This effect is particularly pronounced in Gradient Echo (GRE) sequences, whereas Turbo Spin Echo (TSE) sequences demonstrate higher resilience. The extent of these artifacts depends on several factors, including the type and size of the metallic implant and the strength of the magnetic field. Imaging at 1.5 T produces significantly fewer artifacts than at 3 T, highlighting the impact of field strength on artifact formation.

### 3.2. Artifact Suppression Techniques

To mitigate metal-induced artifacts, the SPACE (Sampling Perfection with Application-optimized Contrasts using different flip angle Evolution) sequence was employed on the Siemens Magnetom Altea 1.5 T system. This sequence, as documented in Table 1 (Section 2.3), demonstrated a reduction in signal voids. However, some residual artifacts were still observed that were not due to the presence of the implant or simulated tumor, but rather due to inappropriate phase encoding settings.

Ideally, these sequences should have been acquired in transverse slices to enhance visualization. The SPACE sequence is a 3D sequence utilizing variable RF flip angles, which helps minimize signal dropouts.

**Figure 3:**
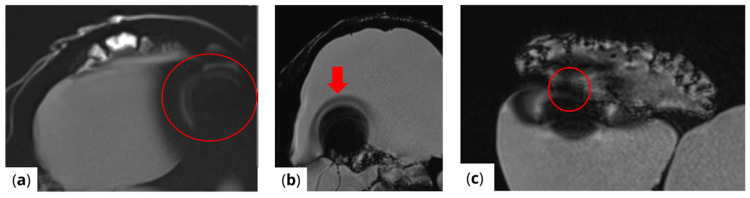
Images illustrating the effect of prolonged echo time (TE) on artifact formation at the interfaces of soft tissue, bone, and air (**a**) t1_starvibe_tra sequence showing signal dropout and distortion near the simulated tumor region, highlighted by the red circle, (**b**) t1_vibe_fs_tra sequence, with the red arrow indicating residual artifact intensity in the vicinity of the implant interface, and (**c**) t2_tse_fs_tra sequence, where the red circle marks susceptibility-induced signal loss at the tumor location.

The extent of MRI signal dropout is also influenced by the echo time (TE); a longer TE exacerbates artifact formation at the interface of soft tissues, bone, and air. This effect is evident in images (Figure 3) obtained using t1_starvibe_tra, t1_vibe_fs_tra, and t2_tse_fs_tra.

### 3.3. Radiofrequency (RF) Artifacts and Signal Homogeneity

**Figure 4:**
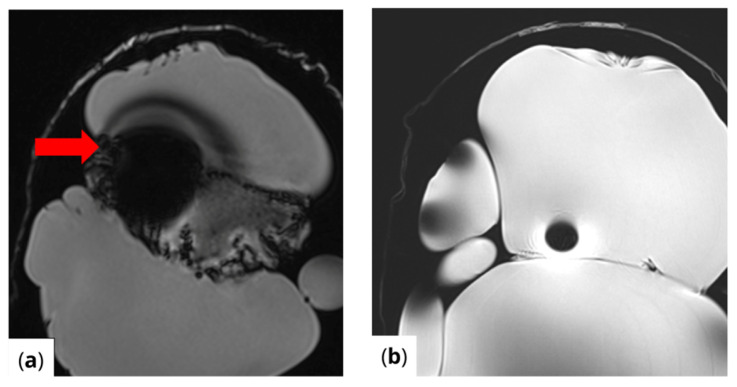
Effects of RF pulse inhomogeneity and high-field interference on signal distribution (**a**) t1_vibe_fs_tra sequence showing pronounced destructive RF interference and signal dropout, indicated by the red arrow (**b**) t2_tse_fs_tra sequence illustrating spatial signal amplification and central inhomogeneity.

RF pulse inhomogeneity can arise due to coil design and signal reception inconsistencies, leading to unwanted spatial signal variations within the image. Differences in local RF flip angles depend on both scanner calibration and RF energy distribution. One disadvantage of high-field MRI (3 T and above) is increased image inhomogeneity caused by RF wave interference within the scanned volume. At higher frequencies, the RF wavelength becomes comparable to the size of scanned structures (e.g., tissues or materials), leading to destructive interference, signal loss in some areas, and signal amplification in others. This effect is clearly visible (Figure 4) in the t1_vibe_fs_tra sequence and t2_tse_fs_tra sequence.

**Figure 5:**
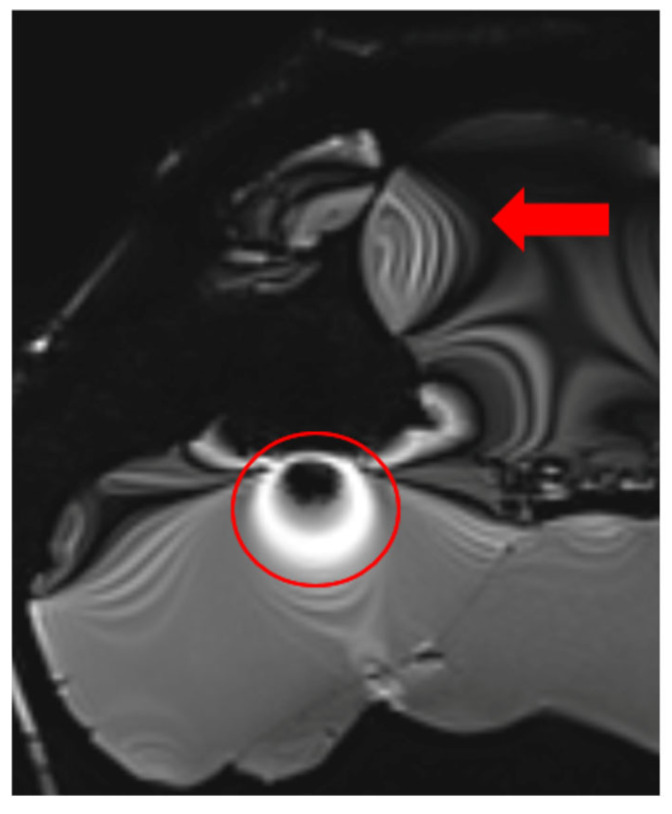
Image illustrating the effects of inaccurate RF pulse calibration on image contrast in the t2_space_flair_sag sequence, with the red circle highlighting signal amplification due to incorrect flip angle settings and the red arrow indicating spatial inhomogeneity caused by improper RF adjustment when scanning small objects.

Additionally, some MRI scanners fail to adjust RF pulse voltages accurately to achieve the intended flip angles, especially when scanning smaller objects than the system anticipates. Before acquiring images, MRI scanners perform an adjustment procedure to calibrate voltage levels for standard 90° and 180° RF pulses. If the calibration is incorrect, all subsequent RF pulses will be inaccurately calculated, leading to improper image contrast. This issue is evident in the t2_space_flair_sag sequence (Figure 5).

### 3.4. Turbo Spin Echo (TSE) for Artifact Reduction

**Figure 6:**
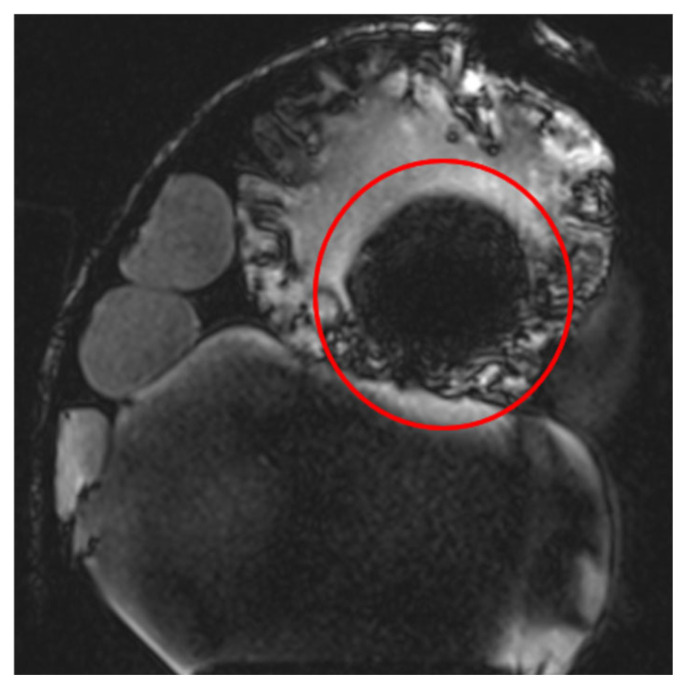
Images demonstrating the impact of varying Echo Train Length (ETL) values in the TSE sequence. Higher ETL values improve local image homogeneity but lead to a reduction in spatial resolution, with extreme ETL values (e.g., ETL = 45) causing noticeable image blurring, as observed in the t2_tse_fs_tra sequence.

The Turbo Spin Echo (TSE) sequence was extensively utilized in this study due to its reduced sensitivity to susceptibility artifacts. This sequence is applicable to multiple contrast types (T1, T2, and Proton Density (PD)) and features an Echo Train Length (ETL) parameter, which controls sequence acceleration. Higher ETL values improve local image homogeneity but can also degrade spatial resolution. At extremely high ETL values (e.g., ETL = 45), a noticeable reduction in image quality is observed, as seen in Figure 6 (t2_tse_fs_tra sequence). Longer ETL values (tens of echoes) result in a blurred spatial resolution, which affects the quality of T2-weighted images.

### 3.5. Fat Saturation Artifacts

In sequences where fat suppression (FS) was applied, regions with higher density structures appeared darker. Spectral Fat Saturation (FATSAT) relies on the difference in resonance frequencies between fat and water. During sequence preparation, an RF pulse selectively excites fat magnetization, flipping it by 90° into the transverse plane.

An applied magnetic gradient then dephases the transverse magnetization of fat, effectively nullifying its signal. Only water magnetization remains, producing a fat-suppressed image.

**Figure 7:**
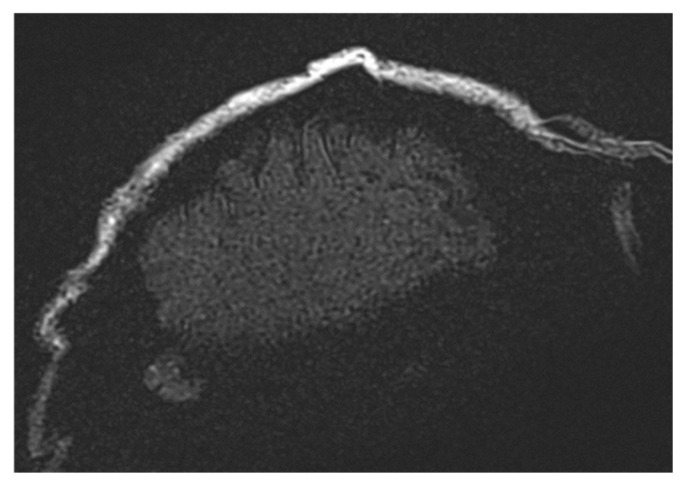
Images illustrating the effects of spectral fat saturation (FATSAT) in MRI sequences, where high-density structures appear darker due to selective fat magnetization suppression. The challenges of achieving uniform fat suppression in large field-of-view (FoV) acquisitions are evident, particularly in the t2_tse_fs_tra sequence.

However, achieving uniform fat suppression requires high magnetic field homogeneity across the entire imaging volume. This becomes particularly challenging in large FoV acquisitions, where maintaining uniformity is nearly impossible. This issue is apparent in Figure 7 (t2_tse_fs_tra sequence).

### 3.6. Optimized Imaging Sequences for Tumor Detection Under Cranial Implants

Table 2 presents the optimized and practically validated MRI sequences designed to achieve the most accurate visualization of tumors or lesions located beneath cranial implants while minimizing artifacts post-reconstruction. These sequences were tested on the Siemens Magnetom Altea 1.5 T scanner (Siemens Healthineers Limited., Bangkok Thailand) to enhance diagnostic quality and improve imaging consistency.

When utilizing the Siemens Magnetom Vida 3 T scanner, multiple sequences were tested. However, due to the constraints imposed by smaller phantom models, certain specialized sequences for lesion visualization could not be acquired. Table 3 includes the sequences that were successfully executed and found to be the most effective for detecting tumors or lesions beneath cranial implants in a higher field strength environment.

## 4. Discussion

The findings of this study align with previous research indicating that MRI artifacts caused by cranial implants vary depending on the material composition, size, and MRI field strength. Several studies have investigated the impacts of different implant materials on MRI imaging, providing valuable insights into artifact formation and potential mitigation strategies.

A comparative study by Krätzig et al., 2020, found that titanium implants exhibit significantly larger MRI artifacts than carbon fiber-reinforced PEEK implants due to their higher magnetic susceptibility [1]. Our results corroborate this, as titanium implants produced more pronounced artifacts, especially at higher magnetic field strengths (3 T). Similarly, Fierens et al., 2023, demonstrated that metal artifact reduction sequences (MARS) can significantly reduce artifacts [2], a finding supported by our study, where Turbo Spin Echo (TSE) sequences with fat suppression minimized distortions.

Another study by Germann et al., 2022 [3], assessed metal-artifact reduction techniques for ultra-high-field (7 T) MRI. The study found that techniques such as View Angle Tilting (VAT) and Slice Encoding for Metal Artifact Correction (SEMAC) significantly improve image quality in the presence of implants [3]. Although our study focused on 1.5 T and 3 T MRI, we observed similar trends where optimized sequences played a critical role in reducing image distortions.

The work of Peschke et al., 2021, emphasized the hazards and imaging challenges posed by metallic implants in MRI, stating that careful sequence selection is essential for reducing signal voids and distortions [5]. Our findings reinforce this assertion, as the improper selection of phase encoding parameters and contrast suppression led to increased artifacts in certain sequences.

Research by Friedrich et al., 2016, and Sunwoo et al., 2018, demonstrated that artifact reduction methods significantly improve visualization near intracranial implants [6,7]. While Sunwoo et al. focused on CT rather than MRI, the principles of material susceptibility affecting image quality remain relevant. Our study found that the practical implementation of optimized imaging sequences led to minimal artifact formation, with artifacts primarily arising due to improper sequence settings rather than the implants themselves.

To reduce MRI artifacts, it is recommended to use implants made from materials with low magnetic susceptibility, such as titanium [8]. The size, surface characteristics, and location of the implant also influence artifact formation, with implants in the orbital region and brainstem area generating higher levels of distortion.

To reduce MRI artifacts associated with cranial implants, it is recommended to use implants made from materials with low magnetic susceptibility, such as titanium [9]. Additionally, the size and surface characteristics of the implant play a crucial role—implants with a smaller diameter and smooth surface generate fewer artifacts [10]. The location of the implant is another determining factor, with implants in the orbital region and brainstem area producing significantly higher levels of distortion [6].

This study provides valuable insights into the field of radiology and MRI imaging, offering critical information on MRI artifact formation following cranial implant placement and strategies for minimizing their effects. These findings can assist clinicians in diagnosing patients with cranial implants and support radiologic technologists in optimizing MRI sequences to improve image quality in clinical practice [11].

Future studies should focus on developing new implant materials with lower magnetic susceptibility to further minimize MRI artifacts, ensuring better compatibility with advanced imaging techniques [12]. Additionally, optimizing MRI sequences specifically tailored for patients with cranial implants could significantly improve image quality and diagnostic accuracy [13]. Another important area of research involves investigating the impact of MRI artifacts on the diagnosis of various neurological disorders, as artifact-related distortions could potentially affect the clinical interpretation [14]. By addressing these aspects, future advancements in MRI technology and implant design can contribute to enhanced diagnostic precision, improved patient outcomes, and more efficient imaging protocols in radiology.

Based on our results, we conclude that the hypothesis was partially confirmed: cranial implants influence MRI artifact formation, with the severity depending on the implant material, size, and magnetic field strength. A higher magnetic field (3 T) induces more pronounced distortions than a lower field (1.5 T). However, optimized imaging sequences, particularly Turbo Spin Echo (TSE) with fat suppression, significantly reduce artifacts and improve lesion visualization [15].

However, it was also evident that artifacts were not solely caused by the presence of implants but were largely influenced by incorrect imaging parameter settings [7]. This suggests that with appropriate sequence selection and optimization, the impact of implants on diagnostic accuracy can be minimized. In conclusion, while cranial implants do generate artifacts, proper imaging techniques can effectively reduce their impacts, confirming only part of the original hypothesis.

One of the main limitations of this study is the potential effect of contrast agent diffusion during the imaging process. Although the contrast agent was enclosed in a balloon catheter system to minimize dispersion, partial diffusion could have occurred over time, potentially affecting the signal intensity and, consequently, artifact evaluation. Additionally, the limited scanning time (a maximum of 20 min per phantom) reduced the risk of contrast dispersion but did not guarantee complete signal stability over prolonged imaging sessions.

Another challenge was the comparison of different imaging angles, with the primary focus placed on the transverse plane. In this study, different imaging angles were analyzed to evaluate their effects on artifact formation and image quality. While the transverse (axial) plane was primarily used due to its clinical importance in neuroimaging, sagittal and coronal orientations were also tested for selected sequences. The results showed that artifact severity varied with the orientation, with the transverse plane often exhibiting more pronounced distortions due to its alignment with the main magnetic field.

In contrast, sagittal and coronal views occasionally reduced artifacts by modifying the phase encoding direction, thereby redistributing susceptibility distortions. To ensure consistency in image acquisition, several external factors were controlled, including standardized phantom positioning, a fixed field of view (FoV), uniform contrast application, and the use of identical MRI hardware (1.5 T and 3 T scanners). These measures helped isolate the impacts of the implant material, imaging sequences, and orientation while minimizing variability. Future research should further investigate optimized angle-dependent imaging strategies to improve diagnostic accuracy in patients with cranial implants and the potential for artifact reduction in sagittal and coronal views.

Finally, although external factors such as temperature and phantom stability were controlled, minor variations in model positioning and magnetic field conditions could have influenced the imaging results. Future research could address these limitations by monitoring contrast stability over longer periods, expanding the analysis of imaging angles, and providing more detailed documentation of the optimization process for MRI sequence selection.

## 5. Conclusions

This study highlights the complex interplay between cranial implants and MRI, emphasizing the challenges posed by implant-induced artifacts on diagnostic accuracy. Using phantom models with titanium and PEEK implants, we were able to systematically evaluate the extent and nature of these artifacts. The findings underscore the importance of selecting implant materials with lower magnetic susceptibility and optimizing imaging protocols, such as employing Turbo Spin Echo (TSE) sequences with fat suppression, to mitigate artifact formation. Furthermore, the use of advanced artifact reduction techniques, including SEMAC and VAT, proved effective at enhancing image quality. These results provide actionable insights for improving MRI protocols, aiding clinicians in obtaining accurate diagnostic data even in the presence of cranial implants. Future research should explore novel implant materials and more robust artifact mitigation strategies to further improve imaging outcomes and patient care.

## Figures and Tables

**Table 1:** Comprehensive summary of the MRI imaging sequences applied to phantom models with cranial implants of varying materials and coverage.

Implant Material	Implant Coverage	Sequence	Slice Thickness [mm]	Sequence Duration [min]	Contrast	Resolution Matrix [mm]
Titanium	Type 1	t2_tse_fs_tra	4	2:11	Strong fat suppression	400 × 544
		t1_starvibe_tra	5	1:45	Fat suppression	256 × 256
		t2_tse_fs_tra	4	3:03	Water suppression	400 × 544
	Type 2	t2_tse_fs_tra	4	2:11	Strong fat suppression	400 × 544
		t1_starvibe_tra	5	2:09	Fat suppression	256 × 256
		t2_tse_fs_tra	4	3:03	Water suppression	400 × 544
	Type 3	t2_tse_fs_tra	4	3:11	Strong fat suppression	400 × 544
		t1_starvibe_tra	5	3:09	Fat suppression	256 × 256
		t1_vibe_fs_tra ^1^	-	2:59	Strong fat suppression	-
PEEK	Type 1	t2_tse_fs_tra	4	2:11	Strong fat suppression	400 × 544
		t1_starvibe_tra	5	2:09	Fat suppression	256 × 256
		t2_tse_fs_tra	4	3:11	Strong fat suppression	400 × 544
	Type 2	t1_starvibe_tra	5	3:09	Fat suppression	256 × 256
		t2_tse_fs_tra	4	3:11	Strong fat suppression	400 × 544
		t1_starvibe_tra	5	3:09	Fat suppression	256 × 256
		t1_space_sag	1	3:03	SPAIR	259 × 259

^1^ The t1_vibe_fs_tra sequence was included in the protocol; however, due to a reduced field of view (FoV), the acquisition was unsuccessful.

**Table 2:** Optimized imaging sequences for the Siemens Magnetom Altea 1.5 T scanner.

Sequence	Slice Thickness [mm]	Sequence Duration [min]	Contrast	TR (Repetition Time) [ms]	TE (Echo Time) [ms]
t2_tse_fs_tra	4	3:11	Strong fat suppression	5190.0	105.00
t1_starvibe_tra	5	3:09	Fat and water suppression	4.4	2.20
t1_space_sag	1	3:03	Fat suppression	700.0	13.00

**Table 3:** Optimized imaging sequences for the Siemens Magnetom Vida 3 T scanner.

Sequence	Slice Thickness [mm]	Sequence Duration [min]	Contrast	TR (Repetition Time) [ms]	TE (Echo Time) [ms]
t2_tse_fs_tra	2	3:11	Strong fat suppression	12,480.0	87.00
t1_vibe_fs_tra	1.1	3:21	Fat and water suppression	6.1	3.57
t1_tse_tra	1	2:09	Fat suppression	12,480.0	87.00

## Data Availability

The original contributions presented in this study are included in the article. Further inquiries can be directed to the corresponding author(s).
